# Biomolecular Network-Based Synergistic Drug Combination Discovery

**DOI:** 10.1155/2016/8518945

**Published:** 2016-11-07

**Authors:** Xiangyi Li, Guangrong Qin, Qingmin Yang, Lanming Chen, Lu Xie

**Affiliations:** ^1^Key Laboratory of Quality and Safety Risk Assessment for Aquatic Products on Storage and Preservation (Shanghai), China Ministry of Agriculture, College of Food Science and Technology, Shanghai Ocean University, 999 Hu Cheng Huan Road, Shanghai 201306, China; ^2^Shanghai Center for Bioinformation Technology, Shanghai Academy of Science and Technology, 1278 Keyuan Road, Shanghai 201203, China

## Abstract

Drug combination is a powerful and promising approach for complex disease therapy such as cancer and cardiovascular disease. However, the number of synergistic drug combinations approved by the Food and Drug Administration is very small. To bridge the gap between urgent need and low yield, researchers have constructed various models to identify synergistic drug combinations. Among these models, biomolecular network-based model is outstanding because of its ability to reflect and illustrate the relationships among drugs, disease-related genes, therapeutic targets, and disease-specific signaling pathways as a system. In this review, we analyzed and classified models for synergistic drug combination prediction in recent decade according to their respective algorithms. Besides, we collected useful resources including databases and analysis tools for synergistic drug combination prediction. It should provide a quick resource for computational biologists who work with network medicine or synergistic drug combination designing.

## 1. Introduction

Complex diseases such as cancer are often caused by a collective of abnormalities of correlated genes or biology processes. The traditional paradigm of “one gene, one drug, one disease” has been challenged by the increasing rate of drug failure and the huge costs of time and money in drug development and research [1, 2]. What is worse, the recurrent drug resistance has significantly reduced the efficacy of the existing drugs [3, 4].

To tackle the problem of drug resistance and reduce the great expenses of time and money in drug discovery, researchers have made great efforts to discover synergistic drug combinations. Synergistic drug combination means that the overall therapeutic effect of the combination is larger than the sum of effects independently caused by individual component [5]. Synergistic drug combination can decrease the drug dosage but increase or maintain the same efficacy to avoid toxicity, minimize, or slow down the development of dug resistance [6]. Inspired by the great benefit of synergistic drug combination, both* in silico* methods and* in vitro* methods have been applied to screen synergistic drug combinations. The most straightforward methods for screening synergistic drug combination are* in vitro* methods.

There are three popular reference models* in vitro* methods, namely, the highest single agent (HSA) model [7], the Loewe additivity model [8], and the Bliss independent model [9]. The difference between these models is their definitions of the noninteraction effect of a drug pair which means the expected additive effect of a drug pair. Specifically, HSA defined the noninteraction effect as the highest monotherapy effect among the individual drug in drug combinations. Loewe additivity model defines the noninteraction effect of a drug pair as if single drug is combined with itself. In contrast, Bliss independence model defines the noninteraction effect of a drug pair as if two drugs work independently. In addition, the integration of the HSA model and the Bliss independence model, called zero interaction potency (ZIP), has been applied to large-scale dose-response matrix experiments [10]. More importantly, Chou proposed a popular algorithm based on the median-effect equation which was encompassed by several complex equations such as Michaelis-Menten, Hill, Henderson-Hasselbalch, and Scatchard equations in biochemistry and biophysics [11, 12]. The core concept of this algorithm, combination index (CI), is an indicator evaluating drug combination interaction effect. CI has been widely used in identification of synergistic drug combination* in vitro*, especially in validation of novel drug combinations from various kinds of computational models. The* in vitro* models mentioned above are all based on drug-treated dose-response curve. Dose-response curve based model can achieve good performance for low-throughput data and be used to validate novel drug combinations. Nevertheless, without involving any molecular level data such as drug-treatment transcriptional expression profile, these models may not help to discover the underlying mechanism of drug synergy [13]. Besides,* in vitro* methods which screen all possible combinations by experimental trials are time and money consuming, and usually only a small number of synergistic drug combinations can be identified.

To address this limitations of* in vitro* methods in identifying synergistic drug combinations,* in silico* methods based on “omics” data have become more and more popular [14–17]. With the explosion of “omics” data in recent years, high-throughput data at various levels related to disease state and drug-treatment have been accumulated rapidly. Also, in the recent decades, protein interactions have been extensively studied, forming comprehensive knowledge background of molecular regulation pathways or networks. In addition, taking the heterogeneity and redundancy of diseases into account, researchers have come to realize that the understanding of mechanism of drug synergistic effect calls for analysis of biology system in a network perspective [1, 18]. Network analysis involves mathematics and computer science into biology and can help to present relationship among molecules in the perspective of network. What is more, network analysis has an advantage of finding newly emerged properties at a network level [19–23]. With the benefit of network analysis, the researchers can make full use of high-throughput data by modeling the interaction among drugs, targets, and diseases, which can definitively promote the discovery of the complex mechanism of drug synergy.

**Figure 1:**
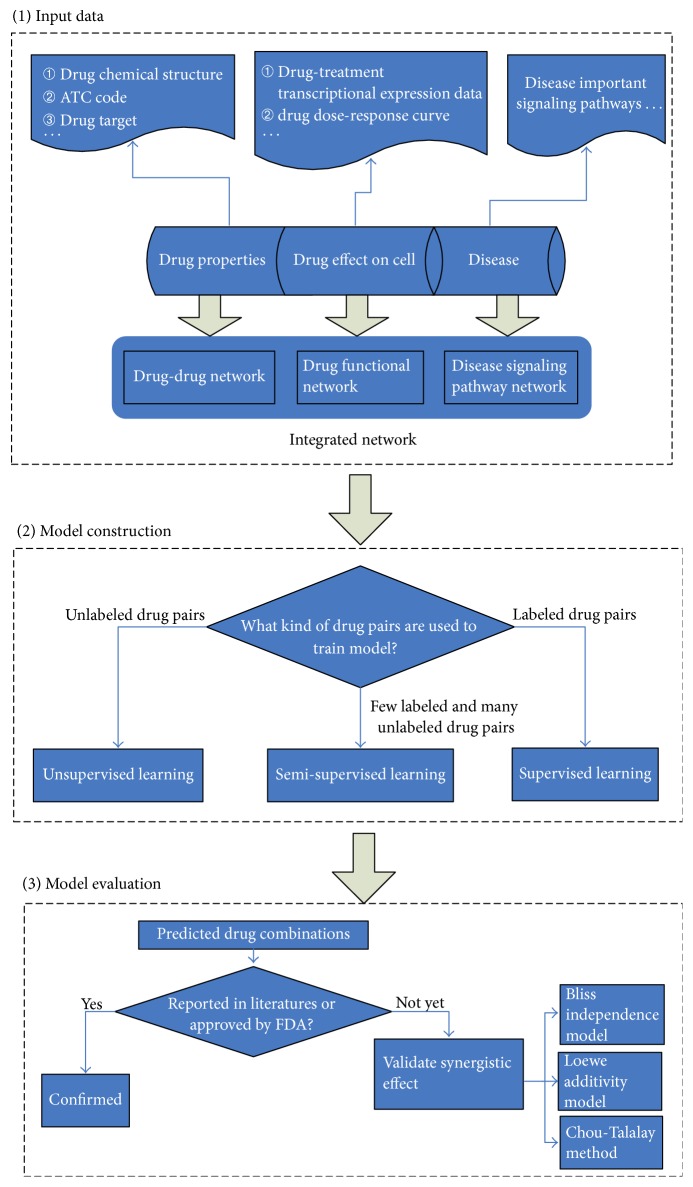
The general work flow of identification of novel synergistic drug combinations based on biomolecular network.

Inspired by the rapid development of biomolecular network-based synergistic drug combination discovery, here we present a brief review on the prediction models of synergistic drug combination. All the models are divided into three classes according to the type of drug pairs used to train the prediction model, namely, unsupervised learning prediction models depending on hypothesis of drug synergy and unlabeled drug pairs, semi-supervised learning prediction models involving few labeled drug pairs, and many unlabeled drug pairs in model training and supervised learning prediction models using labeled drug pairs to train models. Labeled drug pairs denote that effective drug combinations have been approved by FDA or validated by experiments, while unlabeled drug pairs denote drug pairs without synergistic effect evidence. The general work flow of identification of novel synergistic drug combinations based on biomolecular network is shown in Figure 1.

## 2. Important Public Resources for Network Construction in Predicting Synergistic Drug Combinations

Recently, with the rapid development of next generation sequencing (NGS) technologies and rapidly accumulation of “omics” data [24], there are many useful databases and analysis tools for predicting and screening synergistic drug combinations.  Table 1 includes the tools that can be used to identify synergistic drug combinations* in silico*.  Table 2 lists important databases containing many instances of drug combinations for cancers as well as other diseases.  Table 3 lists the popular biology network-related databases that can aid the construction of molecular interaction network.

The Connectivity Map (CMap) collects a genome-wide transcriptional expression data from cultured human cells treated with various bioactive small molecules, providing a bridge to connect drugs and genes [51]. Drug Combination Database (DCDB) contains 1363 drug combinations, providing an important source for model construction and validation [35]. Here, we collected drug combinations from DCDB with each individual component of these drug combinations in CMap database (see supplementary file in Supplementary Material available online at http://dx.doi.org/10.1155/2016/8518945). With these data, the performance of prediction models based on compound/agent-treated transcriptional expression profile can be evaluated without conducting experiments.

## 3. Principles of Identifying Synergistic Drug Combinations Based on Biomolecular Network

The therapy strategy of “one drug, one gene” is not always successful to treat disease because cells can often find alternative ways to compensate the function after a gene or protein is perturbed by drug treatment [52]. Thus to treat these complex diseases, it is beneficial to consider the relationships among drugs, disease-related genes, therapeutic targets, and disease-specific signaling pathways as a system. Known drug combinations provide us a useful resource for predicted novel drug combinations. Taking the known drug combinations into consideration, labeled drug combinations can be used for supervised learning methods. The underlying principle in supervised models is that the more similarity to known drug combinations for a novel drug combination, the more likely it can become a synergistic drug combination. On the other hand, unsupervised models use no labeled drug combination to build and train model and mainly analyze the biological networks perturbed by drug combinations. A network presents the relationships (edges) of a set of entities (nodes). These nodes and edges have various important attributes such as degree, betweenness, and eigenvector centrality. Nodes can denote molecules such as genes, proteins, and drugs. Edges connecting these nodes can represent the interactions such as physical interactions and genetic regulatory interactions [53]. Drug synergy has been reported to be a property largely determined by network topology [54]. Therefore, biomolecular network analysis should provide useful insight into the mechanism of action of drug combinations.

## 4. Biomolecular Network-Based Unsupervised Learning Models for Synergistic Drug Combination Identification

Unsupervised learning models were mainly based on various features of drugs, targets, drug-treated cellular response data, and functional networks following various hypotheses. Drug-treated cellular response data can provide an insight of drug mechanism of action [55]. Transcriptional expression profile is one of the most common cellular response data used to study the mechanism underlying a biological pathway [56] and the biology response of a cell to a certain perturbation [51, 57]. According to different hypotheses, several prediction models have been built.

DrugComboRanker was built based on the hypothesis that effective drug combinations can inhibit major modules of disease signaling networks simultaneously and drugs often have multiple active target genes or proteins [58]. Based on transcriptional expression data from cell lines treated with small molecule compounds in CMap, researchers built a drug functional network and divided the network into numerous drug network communities by a Bayesian nonnegative matrix factorization method [59]. Besides they also constructed a disease-specific signaling network utilizing patients' genomic profile and interactome data. Then they defined a synergy score prioritizing the drug pairs that target on disease-specific signaling network with similar function. Finally, all the drug pairs were ranked in the descend order of the synergy score and the top-ranked drug pairs will be more likely to be synergistic.

Jin et al. built a model called enhanced Petri-net (EPN) to predict the synergistic effect of pairwise drug combinations from genome-wide transcriptional expression data by applying Petri-net to identify drug targeted signaling network [60]. They assumed that there existed at least one molecule that shows the enhanced effect in the pairwise combination compared with the summation of the effect generated by the two drugs individually. They identified synergistic drug combinations by comparing the drug effects from the transcriptional expression data treated by pairwise combination of drugs A and B with those from the corresponding two transcriptional expression data treated by drugs A and B separately.

Wu et al. utilized information of protein interactions, protein-DNA interactions, and signaling pathways to construct a molecular interaction network [61]. Their assumption was that a subnetwork or pathway would be affected in the networked cellular system after a drug was administrated. They built a model to detect the subnetwork perturbed by drug combinations. Based on the molecular interaction network, they defined an interaction score that indicated the gap between drug efficacy effect and side effect. Drug combination whose interaction score for certain subnetwork is higher than that of any individual drug would be recognized as effective drug combination.

Similarly, a model named pathway and pathway interaction (WWI) was based on the assumption that drugs targeting one same pathway or related pathways will be more likely to be synergistic drug combinations [62]. The researchers built two networks, namely, a PPI network based on information from HPRD [44] database and a WWI network based on KEGG database. In WWI network, nodes are the “*Homo sapiens*” pathways, and edges are pathway-pathway interactions. Then for each drug pair they defined a score which indicated the connectivity of pathways perturbed by the individual drug of drug combinations on the WWI network and drug targets on the PPI network. Finally, all the query drug pairs were ranked in descend order of the score and the top-ranked ones would be more likely to be synergetic.

Another group of unsupervised learning prediction models were built according to the DEARM Challenge data. In 2012, the Dialogue on Reverse Engineering Assessment and Methods (DREAM) consortium designed an open competition for researchers all over the world to rank the effect of all 91 pairwise combinations on OCI-LY3 human diffuse large B-cell lymphoma (DLBCL) cell line from the most synergistic to the most antagonistic [63]. This project generated transcriptional expression data only for samples treated by individual drug, dose-response curves for viability of OCI-LY3 cells following perturbation with 14 distinct compounds and baseline genetic profile of the OCI-LY3 cell line. Among all the 31 groups taking part in the project, three of them performed significantly better than random guess. All of the three groups developed their models based on their assumptions about drug synergy, such as assumption that changes in gene expression after drug perturbations could be used to predicted these drug interaction effect [55] or the correlation of differential expression genes (DEGs) after two drugs perturbations would reflect the possibility of drug synergistic effect [64]. Although the final result of this project was modest, the challenge gives us reasons to hope for powerful methods to identify effective drug combinations in the future [65].

Among all of those 31 groups participating in the project, Drug-Induced Genomic Residual Effect (DIGRE) model achieved the best performance on prediction of synergistic drug combinations [66]. DIGRE was developed based on the hypothesis that, for two drugs used sequentially, the first drug would change the transcriptome of the treated cell and thus modulate the effect of the other one. Firstly, the researchers constructed a gene-gene interaction network based on KEGG pathways. Based on the network, DIGRE required transcriptional expression profiles and dose-response curves provided by the DREAM Challenge as the input data. Then drug similarity score of the drug pair was computed by accounting for DEGs of each drug, including common DEGs and upstream and downstream genes in the selected pathways. Finally, all 91 drug pairs were ranked in descend order based on the combinatorial effect score which were computed based on corresponding drug response curve and drug similarity score.

From above, we can see that drug-treated transcriptional expression data is commonly used in unsupervised learning prediction models because of its informative properties, such as reflecting the mechanism of drug action underlying a biological pathway. However, there is no standard rule to process transcriptional expression data. Thus, selection of significant differential expression genes is largely depended on individual researchers and significant gene lists can be quite diverse according to different algorithms. For models based on transcriptional expression data, different networks will be constructed by different researchers depending on diverse processing methods of the same microarray data, which can lead to difficulty in the final interpretation [31].

## 5. Biomolecular Network-Based Semi-Supervised Learning Models for Synergistic Drug Combination Identification

Traditional classifier uses only labeled data to train the prediction model. However, it is both time and money consuming to collect labeled data by experts. Semi-supervised learning solves this problem by using large amount of unlabeled data together with a limited number of labeled data [67]. To the best of our knowledge, the number of approved drug combinations is still much less than drug combinations without synergistic effect evidence, so semi-supervised learning can solve this problem.

Sun et al. constructed a model called Ranking-system of Anticancer Synergy (RACS) based on semi-supervised learning which was used to rank drug pairs according to their similarity to the labeled samples in a specified multifeature space [68]. Firstly, they performed feature selection to identify significantly different features between labeled samples and the unlabeled samples. Some interesting features had been identified, such as drug target distance in PPI network and the proportion of unrelated pathways regulated by the targets of the two agents. Then all the drug pairs (i.e., labeled and unlabeled samples) were represented by a vector of the selected features (mentioned in the previous step). Finally, they incorporated a manifold ranking algorithm with semi-supervised learning method to enrich the labeled pairs at the top of the drug pair list [69]. To evaluate the performance of RACS in test dataset, the researchers applied RACS to data provided by the above mentioned DREAM Challenge project [49]. It impressively achieved greater progress than that of the best model, DIGRE. However, despite the complexity of the manifold ranking algorithm and some other complex mathematics methods used in RACS, it also largely relied on the known drug targets to calculate the average distance between the target proteins of the two agents in the context of PPI network. So far, a part of compound targets are still unknown; thus this will limit the application of RACS in synergistic drug combination identification.

Chen et al. developed an algorithm termed Network-based Laplacian regularized Least Square Synergistic drug combination prediction (NLLSS) based on their observation that principal drugs which obtain synergistic effect with similar adjuvant drugs are often similar [70], where principal drug means that the drug in synergistic drug combination shows activity in disease treatment and adjuvant drug means drug in synergistic drug combination shows no effect on disease treatment. NLLSS developed a classifier based on the framework of Laplacian Regularized Least Square (LapRLS) which is an popular semi-supervised learning algorithm [71]. Firstly, researchers computed drug similarity for principle drugs and adjuvant drugs, depending on several integrated information such as known synergistic drug combinations, drug combinations without known synergistic evidences, drug target interactions, and drug chemical structures. Finally, a score used to assess synergistic probability of a drug combination can be obtained depending on the result from previous step.

As can be seen from above, RACS and NLLSS share common features. Firstly, they both have a small number of labeled data, such as 26 labeled data compared with 502 unlabeled data for RACS training set and 75 labeled data compared with 4079 unlabeled data for NLLSS training set. Secondly, they were developed based on known and complex machine learning algorithms for manifold regularization which is a technique for using the shape of a dataset to constrain the functions that should be learned on that dataset [71].

## 6. Biomolecular Network-Based Supervised Learning Models for Synergistic Drug Combination Identification

Supervised learning is a machine learning algorithm of inferring a function from training data. The training data consists of a set of training samples which consist of an input object and a relevant label. Then with the inferred function, new sample can be labeled [72]. Thus with proper amount of known synergistic drug combinations as a training set, researchers can get a learned function by supervised learning which can identify candidate synergistic drug combinations.

Zhao et al. developed a model based on features of US Food and Drug Administration (FDA) approved drug combinations including drug features such as drug target proteins and corresponding downstream pathways, medical indication areas, therapeutic effects as represented in the Anatomical Therapeutic Chemical (ATC) Classification System, and side effects [13]. They performed 5-fold cross-validation on the above mentioned drug combinations to evaluate the performance of these features. Then the learned model was revised after deleting two weakly predictive features. Finally, drug pairs between marketed drugs from FDA orange book were applied to evaluate the performance of the model.

Xu et al. proposed a model called Drug Combination Predictor (DCPred) [73]. With the effective drug combinations collected from DCDB, they built a so-called drug-cocktail network which contained 215 nodes (i.e., unique drug) and 239 edges (i.e., known synergistic effect). Their hypothesis was that two drugs which shared large number of common drugs in drug-cocktail network would be more likely to be effective drug combinations. They found that, compared with drugs in random combination network, drugs in drug-cocktail network tended to have more therapeutic effects and more interaction partners. Based on these two topological features of drug combinations, they built a function which required parameters such as number of common neighbors of two drugs in drug-cocktail network and unique neighbors of individual drug to predict the probability for a drug pair to be an effective drug combination.

Similarly, Li et al. proposed another model called probability ensemble approach (PEA) based on drug-based similarity features including drug chemical structure, ATC code, target side effect, and target-based similarity features include target sequence, target-target interaction in PPI, and Gene Ontology (GO) semantic [74]. The six features for every drug pair were combined using a Bayesian network to calculate a likelihood ratio (LR) which can be used to the estimate of the similarity to known drug pair interaction [75]. A raw score for a query drug pair was defined by summing LRs to all the known drug pairs in each set (i.e., effective drug combinations or undesirable drug-drug interactions), which is further converted to *p* value based on random distribution. After performance evaluation of PEA using 10-fold cross-validation scheme accompanied with the receiver operating characteristic (ROC) curve analysis, integrative analysis of side-beneficial effects for drug combinations, external literature validation, and experimental validation, tens of effective drug combinations were confirmed.

Chandrasekaran et al. developed a computational model entitled INferring Drug Interactions using chemo-Genomics and Orthology (INDIGO) which used chemogenomics data to predict antibiotic drug combinations [76]. The core of INDIGO is a machine learning algorithm called random forest. To train INDIGO, the researchers firstly performed experimental measurement of 105 interactions (*C*_15_^2^ = 105) among 15 drugs. Then drug interaction data measuring synergistic and antagonistic effect together with chemogenomics data were put into INDIGO as training data. INDIGO only requires chemogenomics data of individual drugs to output novel drug combinations.

The supervised learning models tend to take advantage of drug property information like drug target, ATC code, and chemical structure, while drug synergy is strongly context-dependent, disease type [77] and drug dosages [60] can also modulate the efficacy of drug combinations. Therefore, future supervised learning models should take drug properties as well as drug-treated information into consideration to improve performance of prediction model.

## 7. Conclusions

With the explosive growth of high-throughput data,* in silico* modeling for synergistic drug combination represents both an opportunity and a challenge for medicine research. Combined with knowledge of mathematics, computer science, and biology, analysis of complex molecule interactions based on biomolecular networks can greatly accelerate the discovery of synergistic drug combinations [78]. To build a model with great prediction performance, intricate mathematics method is necessary to simulate the interactions between molecules in the complex biology system. For validation of the predicted novel drug combinations,* in vitro* methods should be used to get dose-response curves. Finally, either Chou-Talalay method or reference models such as Loewe additivity model and Bliss independent model can be used to determine the effect of drug combination (i.e., synergistic, antagonistic or additive).

Based on the review above, we can see that the number of biomolecular network-based unsupervised learning models is much bigger than that of semi-supervised learning or supervised learning. The possible reason is that only small number of drug combinations has been approved by regulatory agency, which limits the use of machine learning methods such as semi-supervised learning method and supervised learning method. Several significantly different features between synergistic drug combinations and random drug combinations have been identified. The average shortest distance in PPI network of targets between synergistic drug combinations is significantly smaller than that in random drug combinations [68, 73, 79]. Also, dissimilarity of drug chemical structure for individual drug in drug combination is significantly associated with drug synergistic effect [80].

There are several limitations in effectively applying these network-based models. Firstly, synergistic drug combination modeling utilizing high-throughput data based on biomolecular network is in its infancy and most of these models have not been fully validated in practice. Secondly, despite the complexity of these prediction models, the massive noise of high-throughput data at different levels from different contributors can play an import role in model construction and in turn lower the performance of the prediction model. For instance, prediction models can perform very well in one test set, while poor result may be achieved when the model is applied to another test set. For example, Sun et al. applied the transcriptional expression profiles and target information of drug treatment on the human lung adenocarcinoma cell line A549 to RACS [68]. Results showed that only two drug combinations are synergetic ranked the top 10%; however drug combinations ranked the first and second are both antagonistic. Thirdly, networks such as PPI network, gene-protein interaction network, and drug target interaction network all have been considered in models mentioned above except metabolism network. Drug metabolism processes such as drug absorption and transportation are very important in disease treatment [81]. For example, one of the most important reasons for drug resistance is the overexpression of the P-glycoprotein (P-gp) [82], a drug efflux protein expressed by ABCB1 which is from ATP-binding cassette (ABC) family [83–85], and it has been reported that inhibition of P-gp can enhance the drug efficacy [86–90]. Thus, we deduced that drugs inhibiting efflux genes (e.g., ABC family genes) or activating drug influx genes (e.g., solute carrier transporter genes [91]) can make a contribution to drug synergistic effect. However, few prediction models took drug metabolism processes into consideration.

Future models for synergistic drug combination prediction should pay more attention to incorporating comprehensive information including disease signaling pathways and drug targeting pathways as well as drug metabolism processes such as drug absorption, transportation, metabolism, and clearance.

## Supplementary Material

Pairwise drug combinations in Drug Combination Database (DCDB), components of which are both in Connectivity Map.

## Figures and Tables

**Table 1:** Tools used to analyze drug combination data.

Tool name	Tool type	Reference model(s)	Input data	Brief description
CompuSyn [25]	Free software	Loewe additivity model	Dose-response data	CompuSyn only allows for manual input of one drug combination at a time
Synergyfinder [26]	R package	HSA, Loewe additivity, Bliss independence	Dose-response data	Synergyfinder is implementations for all the popular synergy scoring models for drug combinations, including HAS, Loewe, Bliss, and ZIP [10]
Mixlow [27]	R package	A nonlinear mixed-effects model	Dose-response data	Mixlow used a nonlinear mixed-effects model to estimate parameters of dose-response curves and required experimental design where the ratio of two drugs in a combination is fixed
COMBIA [28]	R package	Bliss independence, Loewe additivity	Data from wet-lab experimental	Data from wet-lab experimental platforms can be directly used
MacSynergyII [29]	Free software	Bliss independence	Dose-response data	MacSynergy II is essentially an Excel file and it scales the input data to %inhibition using positive and negative controls
Combenefit [30]	Free software	HSA, Loewe additivity, Bliss independence	Dose-response data	Combenefit has advanced graphical capabilities and can be applied to model-based quantification of drug combinations in single and high-throughput settings
Combinatorial Drug Assembler [31] (http://cda.i-pharm.org/)	Free web app implementation	None	Disease-related signaling pathway components	CDA performs expression pattern matching between input gene sets and 6,100 molecule-treated expression profiles of the connectivity map to list up best pattern matching single drugs/combinatorial drug pairs
Synergy Maps [32] (http://richlewis42.github.io/synergy-maps/)	Free web app implementation	None	Drugs or drug combinations in two datasets [25, 26]	Synergy Maps can simultaneously represent individual compound properties and their interactions
DT-Web [33] (http://alpha.dmi.unict.it/dtweb/)	Free web app implementation	None	The name or the accession number of a drug/target	A web-based application for drug-target interaction and drug combination prediction
TIMMA-R [34]	R package	Logic-based network	Drugs' polypharmacological profiles and drug sensitivity profiles from a given cancer cell line	TIMMA-R predicts the effects of drug combinations based on their binary drug target interactions and single-drug sensitivity profiles

**Table 2:** Integrated drug combination databases.

Database	URL
DCDB [35]	http://www.cls.zju.edu.cn/dcdb/
TTD [36]	http://bidd.nus.edu.sg/group/cjttd/
TCM [37]	http://tcm.cmu.edu.tw/
ASDCD [38]	http://asdcd.amss.ac.cn/

**Table 3:** Important biology network-related databases.

Database	URL	Data type
STRING [39]	http://string-db.org/	Protein-protein interactions
Reactome [40]	http://www.reactome.org/	human biological processes
KEGG [41]	http://www.genome.jp/kegg/	Pathway, disease, drug
BioGRID [42]	https://wiki.thebiogrid.org/	PPI/genetic interaction
STITCH [43]	http://stitch.embl.de/	Chemical-protein interaction
HPRD [44]	http://hprd.org/	Protein-protein interaction (PPI)
DIP [45]	http://dip.doe-mbi.ucla.edu/dip/Main.cgi	PPI
IntAct [46]	http://www.ebi.ac.uk/intact/	Molecular interaction
WikiPathways [47]	http://www.wikipathways.org/index.php/WikiPathways	Biological pathways
TRED [48]	https://cb.utdallas.edu/cgi-bin/TRED/tred.cgi?process=home	TF-gene interaction
InterDom [49]	http://interdom.i2r.a-star.edu.sg/	Domain interaction
SignaLink [50]	http://signalink.org/	Signaling pathways
